# 3D patient imaging and retrieval analysis help understand the clinical importance of rotation in knee replacements

**DOI:** 10.1007/s00167-018-4891-9

**Published:** 2018-03-08

**Authors:** Arianna Cerquiglini, Johann Henckel, Harry Hothi, Niccoló Rotigliano, Michael T. Hirschmann, Alister J. Hart

**Affiliations:** 1 Institute of Orthopaedics and Musculoskeletal Science, University College London and the Royal National Orthopaedic Hospital, Stanmore, UK; 2 Department of Orthopaedic Surgery and Traumatology, Kantonsspital Baselland (Bruderholz, Liestal, Laufen), 4101 Bruderholz, Switzerland

**Keywords:** Total knee replacement, Total knee arthroplasty, Implant orientation, Malalignment, 3D computed tomography, Polyethylene surface damage

## Abstract

**Purpose:**

The purpose of the present study was to correlate highly accurate CT measurements of pre-revision total knee arthroplasty (TKA) implant position with findings of retrieval analysis post-revision, to understand the clinical relevance of TKA orientation.

**Methods:**

This study involved 53 retrieved TKA implants with pre-revision 3D-CT scans used to determine coronal (varus–valgus), sagittal (tibial slope) and rotational (internal rotation–external rotation) TKA orientation as well as tibiofemoral leg axis. Differences between femoral and tibial angles to describe the "relative rotational mismatch" were also calculated. All tibial inserts were forensically analyzed using the Hood score. Statistical analysis was performed to investigate correlations between TKA component orientation and surface damage (*p* < 0.05).

**Results:**

Femoral components were found to have axial rotations mainly within ± 3° (68%), whilst 45% of the tibial components and 66% of the relative rotational mismatches were > 3° and < − 3°, respectively. The majority of femoral and tibial components (87% in both cases), as well as the femorotibial angle (70%), showed coronal orientations within ± 3°. The 64% of the tibial components showed posterior tibial slopes out of both the 0°–3° and 5°–7° ranges. There was a significant correlation between tibial slope and damage score on polyethylene tibial inserts (*r* = 0.2856; *p* = 0.0382) as well as a significant correlation between implants’ position in the axial plane and damage score on polyethylene tibial inserts (*r* = 0.6537, *p* = 0.0240).

**Conclusions:**

This is the first study to use accurate measurements from pre-revision 3DCT to compare tibial and femoral orientation in all three planes with retrieval findings in total knee replacements. A significant correlation between implant position and polyethylene surface damage was found. These results showed the importance of optimizing component position to minimize polyethylene damage. Further analysis involving more accurate polyethylene wear measurements are fundamental to fully understand the role of components’ orientation in TKAs.

## Introduction

Total knee arthroplasty (TKA) is a common intervention with an excellent survival rate; however, up to 20% of patients have reported poor outcomes, leading to revision [1]. This phenomenon has global dimensions: the demand for revision of TKAs is projected to grow by 601% between 2005 and 2030 in the United States [2] and the same trend is also expected in Europe and Asia Pacific [3, 4].

The reasons for TKA failure are multifactorial and influenced by surgical, implant and patient factors. The most commonly reported reasons for revision are aseptic loosening, pain, infection, instability, stiffness, polyethylene wear, malposition, patellofemoral problems and dislocation/subluxation [1, 5–9].

A suboptimal TKA position of femoral and tibial components contributes to poor outcomes, premature polyethylene wear and “unexplained” painful TKA [7, 8, 10–15]. It is well established that excessive internal rotation of the femoral component in the axial plane leads to patellar maltracking, anterior knee pain and flexion instability [7, 10–12]. Some studies also found a significant correlation between malposition in the coronal plane and aseptic loosening of the implant, due to a higher amount of polyethylene wear caused by abnormal force distributions [16–18]. Suboptimal flexion was found in cruciate retaining TKA with reduced posterior tibial slope [16].

Findings from retrieval studies of failed TKA implants provide valuable information on the location and potential mechanisms for TKA component damage in vivo [17, 19]. Several papers have suggested that the wear pattern is associated with different clinical and mechanical factors, such as component position, orientation and alignment [13–15, 18, 20–25]. For example, it has been shown that there is often more medial wear on tibial polyethylene bearings in well-positioned knees [7], and changes in orientation are known to influence this (increased medial wear in varus knees and greater lateral in valgus knees) [18, 25]. Malrotation might cause abnormal stresses and premature wear of the polyethylene components, followed by peri-prosthetic and implant loosening [7, 8, 10, 11, 18, 19]. However, the relationship between implant orientation and wear patterns in retrieved knee prostheses is still poorly understood.

The aim of this study was, for the first time, to correlate highly accurate 3D-CT measurements of pre-revision TKA position, provided by an innovative 3D imaging technique [8], with retrieval analysis findings post-revision to better understand implant orientation effects on TKA.

## Materials and methods

**Fig. 1:**
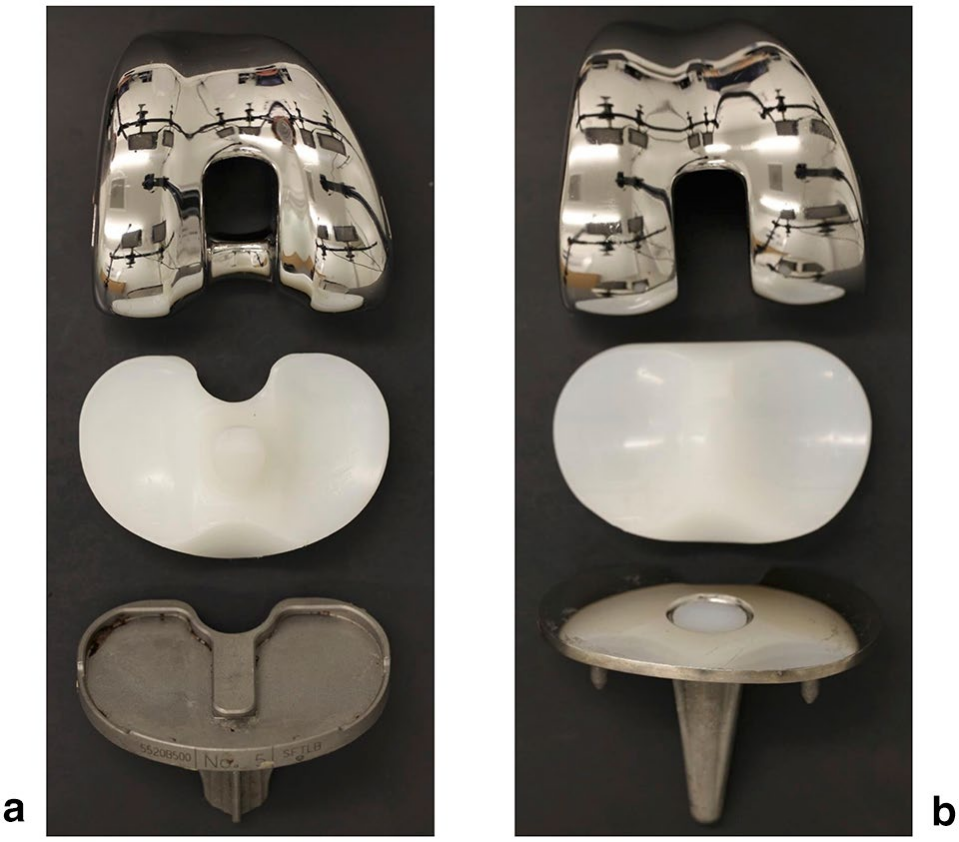
Examples of contemporary retrieved knee implants. **a** Posterior stabilized (*PS*) TKA with fix bearing, **b** cruciate retaining (*CR*) TKA with rotating bearing

This was a retrieval study involving 53 contemporary TKAs that had been consecutively revised at a single institution. The implants were revised from 30 female and 23 male patients with a median (range) age of 62 (42–78) years and a median time to revision of 38 (5–162) months. The reasons for revision were instability (*n* = 26), malposition (*n* = 11), patella maltracking (*n* = 7), aseptic loosening (*n* = 3), pain (*n* = 2), stiffness (*n* = 2), infection (*n* = 1) and arthrofibrosis (*n* = 1). The retrieved TKAs consisted of 40 cruciate retaining (CR) and 13 posterior stabilized (PS) TKA (Fig. 1). Table 1 summarizes the TKA specifications and patient demographics for each case.

**Table 1:** Implant and patient demographic

Case number	Design	Manufacturer	Patient age	Gender	Time to revision [months]	Reason for revision
1	NexGen LCCK, PS	Zimmer	69	M	29	Infection
2	Innex, CR	Zimmer	65	F	71	Instability
3	PFC Sigma, CR	DePuy	69	M	15	Aseptic loosening
4	PFC Sigma, CR	DePuy	66	M	53	Malposition
5	Triathlon, PS	Stryker	53	F	79	Instability
6	Natural Knee II, CR	Zimmer	69	M	36	Instability
7	Triathlon, PS	Stryker	75	M	99	Aseptic loosening
8	Triathlon, CR	Stryker	66	M	72	Instability
9	BalanSys, CR	Mathys	61	F	40	Patella maltracking
10	Natural Knee II, CR	Zimmer	71	F	90	Patella maltracking
11	PFC Sigma, CR	DePuy	46	F	20	Pain
12	TC PLUS, CR	Smith&Nephew	62	M	10	Instability
13	Type LCS, CR	DePuy	46	M	120	Instability
14	ATTUNE, PS	DePuy	68	F	15	Instability
15	Signature, PS	Zimmer	64	M	10	Malposition
16	NexGen, PS	Zimmer	51	F	6	Pain
17	Type LCS, CR	DePuy	51	F	22	Instability
18	Synthes-LCS, CR	DePuy	71	F	24	Patella maltracking
19	NexGen, PS	Zimmer	48	M	36	Instability
20	Synthes-LCS, CR	DePuy	64	F	162	Instability
21	Persona, CR	Zimmer	57	M	60	Patella maltracking
22	Type LCS, CR	DePuy	70	F	12	Instability
23	Journey, CR	Smith&Nephew	48	M	21	Instability
24	PFC Sigma, PS	DePuy	53	F	45	Malposition
25	BalanSys, CR	Mathys	62	F	18	Instability
26	ATTUNE, PS	DePuy	78	F	5	Instability
27	Triathlon, CR	Stryker	60	M	91	Instability
28	BalanSys, CR	Mathys	56	M	25	Instability
29	BalanSys, CR	Mathys	53	F	22	Instability
30	PFC Sigma, CR	DePuy	62	F	60	Instability
31	Innex, CR	Zimmer	66	F	13	Malposition
32	TC PLUS, CR	Smith&Nephew	59	M	101	Stiffness
33	BalanSys, CR	Mathys	42	F	51	Malposition
34	Gemini, PS	Link	72	F	58	Malposition
35	PFC Sigma, CR	DePuy	73	F	37	Malposition
36	PFC Sigma, PS	DePuy	72	F	19	Stiffness
37	PFC Sigma, PS	DePuy	63	F	61	Aseptic loosening
38	Triathlon, CR	Stryker	49	F	17	Instability
39	BalanSys, CR	Mathys	66	M	77	Instability
40	Colombus, CR	Aesculap	59	F	60	Patella maltracking
41	BalanSys, CR	Mathys	46	M	46	Malposition
42	PFC Sigma, CR	DePuy	70	M	64	Instability
43	ATTUNE, CR	DePuy	67	F	13	Patella maltracking
44	ATTUNE, CR	DePuy	64	F	22	Instability
45	Synthes-LCS, CR	DePuy	62	F	24	Instability
46	BalanSys, CR	Mathys	68	M	156	Malposition
47	Natural Knee II, CR	Zimmer	59	M	15	Malposition
48	BalanSys, PS	Mathys	55	F	100	Instability
49	Natural Knee II, CR	Zimmer	74	M	38	Malposition
50	Natural Knee II, CR	Zimmer	56	M	79	Malposition
51	PFC Sigma, CR	DePuy	60	M	101	Instability
52	PFC Sigma, CR	DePuy	61	F	10	Instability
53	Triathlon, CR	Stryker	62	F	66	Patella maltracking

All investigations were conducted in conformity with ethical principles of research, that informed consent for participation in the study was obtained and that institutional approval of the human protocol for this investigation was obtained.

### 3D computed tomography (CT) position

**Fig. 2:**
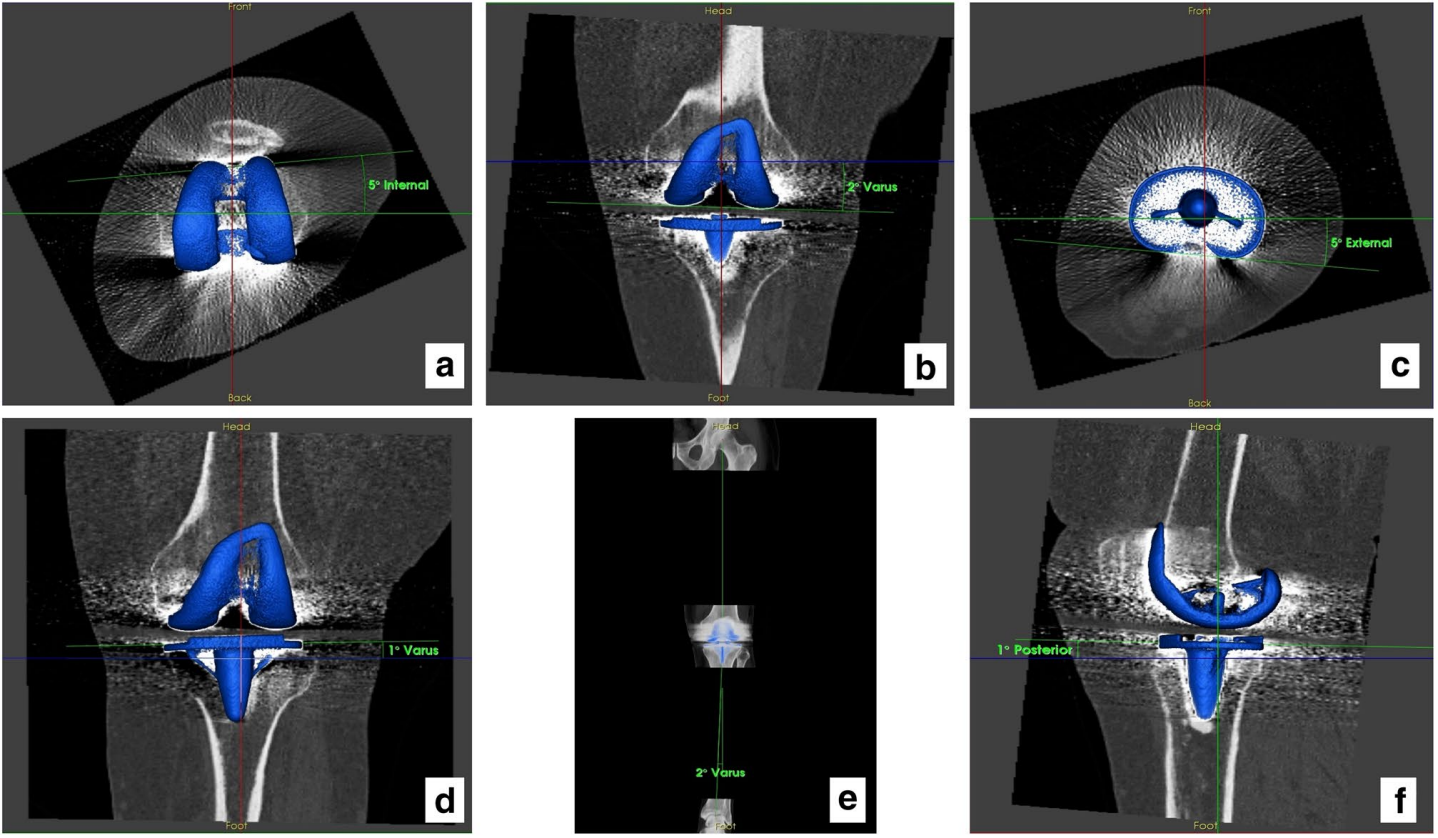
A 3D CT image from a patient illustrating components’ positions. **a** View from the transverse plane showing internal rotation of the femoral component; **b** view from the coronal plane showing varus angle on the femoral component; **c** view from the transverse plane showing external rotation of the tibial component; **d** view from the coronal plane showing varus angle of the tibial component; **e** view from the transverse plane showing tibiofemoral angle; **f** view from the sagittal plane showing posterior slope of the tibial component

Pre-revision CT scans of both the femoral and tibial components from each patient were taken using the imperial CT protocol, which obtains the relevant bony landmarks (hip–knee–ankle) and includes specific metal artefact reduction sequences [8, 26]. From the CT images, the reference axes (anatomical and mechanical) were defined, the images standardized and the angles computed, to provide the following positioning measurements: (1) axial rotation in the transverse plane of both the femoral and tibial components, with reference to the transepicondylar and anteroposterior axes, respectively, (2) the degree of varus/valgus of single components and the tibiofemoral angle in the coronal plane and (3) the tibial component slope in the sagittal plane, Fig. 2.

Differences between femoral and tibial axial rotation angles to describe the relative rotational mismatch [27] between these two components in the transverse plane were also computed; the measured angles were used to estimate the magnitude of this mismatch, while the sign explained the relative position between the femoral and the tibial components (negative values meant that the femoral component was internally rotated compared to the tibial one; positive values meant that the femoral component was externally rotated compared to the tibial one).

**Fig. 3:**
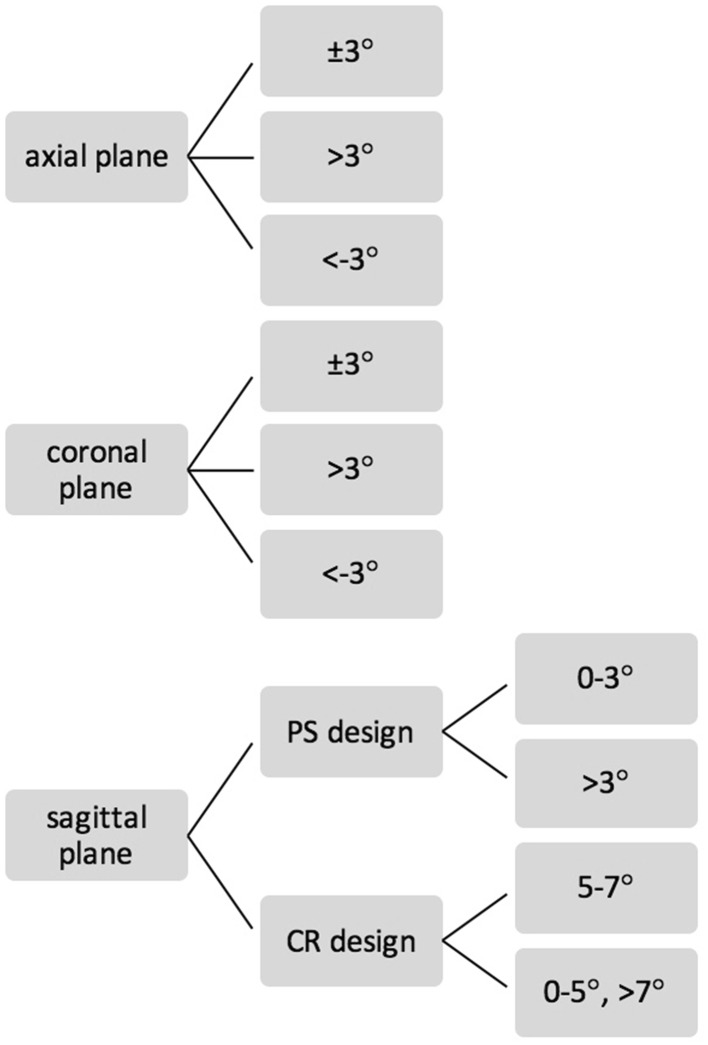
Classification of the orientations in the three planes (axial, coronal and sagittal orientations)

The measured values were classified into three ranges (Fig. 3), in agreement with surgical standard aims [28, 29].

### Explant preparation

All implants were decontaminated using 10% formalin solution. The polyethylene components were then stored in a freezer at − 18 °C, to minimise the oxidation process.

### Analysis of polyethylene surfaces

**Fig. 4:**
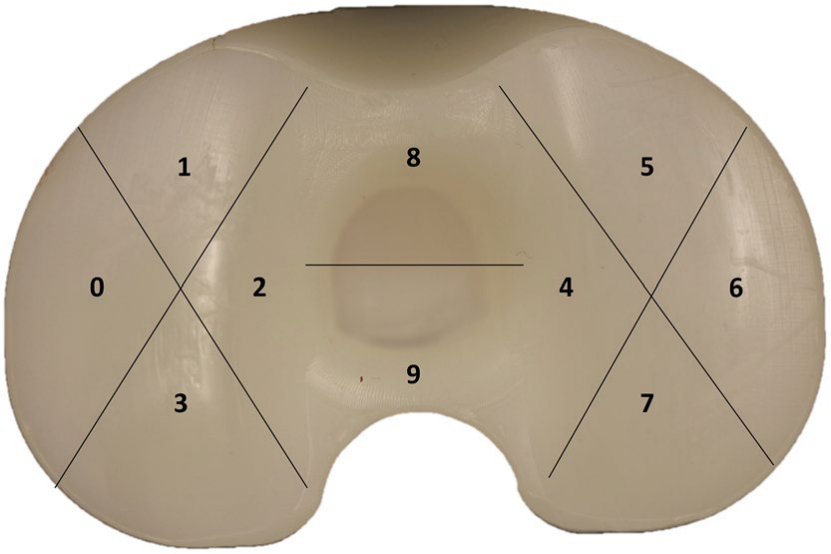
Surface divisions on the articular surface of the tibial insert, showing 10 sections (from 0 to 9) defined by Hood [30]

The tibial polyethylene inserts (*n* = 53) were forensically analyzed using a Keyence VHX-700F series (Keyence Co., Japan) digital microscope, with magnification from 50× up to 200×. On the articular surface, both the medial and lateral sides were divided into four sections, while the central part of the insert into two sections (Fig. 4). Each of the 10 total sections were analyzed using the Hood score, according to the presence and severity of seven modes of surface degradation (surface deformation, pitting, embedded debris, scratching, burnishing, abrasion and delamination) [30], Table 2. The maximum damage grade was 21 for a single section (grade 3 for each of the seven damage modes) and 210 for the entire surface (grade 3 for each of the seven damage modes for each of the 10 sections). Grading was performed by two different examiners. In case of disagreement, the examiners discussed the results together, to agree a final grade.

**Table 2:** Damage-grading criteria used to assess polyethylene component surface, according to the Hood score [30]

	Polyethylene surfaces
Modes of surface degradation	Deformation, pitting, embedded debris, scratching, burnishing, abrasion, delamination
Score	
0	Not visible
1	< 10% of surface
2	10–50% of surface
3	> 50% of surface
Maximum section score	21
Maximum total score	210

### Statistical analysis

**Fig. 5:**
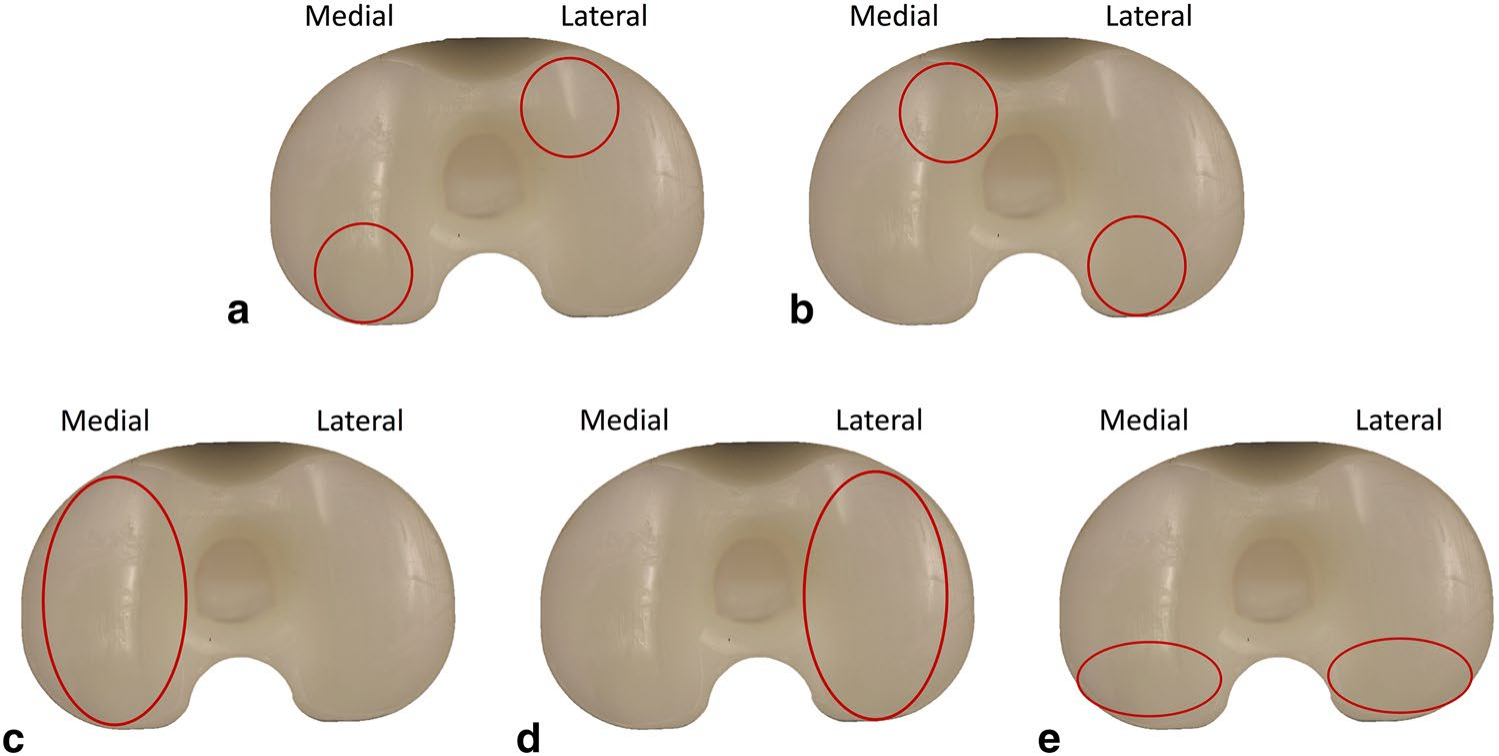
Localisation of expected wear (on a right knee) according to implant orientations. **a** An internal relative rotational mismatch is expected to increase the damage on the posterior compartment in the medial side and on the anterior compartment in the lateral side; **b** an external relative rotational mismatch is expected to increase the damage on the posterior compartment in the lateral side and on the anterior compartment in the medial side; **c** a varus tibiofemoral angle is expected to increase the damage on the medial compartment; **d** a valgus tibiofemoral angle is expected to increase the damage on the lateral compartment; **e** posterior slope is expected to decrease the damage on posterior compartments

Statistical analysis was performed to determine if there were any significant correlations between orientation of components and expected location of the surface damage, described in the literature [18, 20, 24, 25]. Potential associations between (1) internal relative rotational mismatch and the amount of damage on the posterior compartment in the medial side and on the anterior compartment in the lateral side, Fig. 5a; (2) external relative rotational mismatch and the amount of damage on the posterior compartment in the lateral side and on the anterior compartment in the medial side, Fig. 5b; (3) varus tibiofemoral angle and the amount of damage on the medial compartment, Fig. 5c; (4) valgus tibiofemoral angle and the amount of damage on the lateral compartment, Fig. 5d; (5) posterior slope and amount of damage on the posterior compartments, Fig. 5e were analyzed (two-tailed non-parametric Spearman correlation, *p* value < 0.05 was considered as significant).

Potential significant differences in the total Hood score between optimal and suboptimal orientation were also investigated (Mann–Whitney test, *p* value < 0.05 was considered as significant).

Separate statistical analyses on implants revised for malposition were performed and significant differences between implants with short (≤ 2 years) and long (> 2 years) implantation time were also investigated (Mann–Whitney test, *p* value < 0.05 was considered as significant). All statistical analyses were performed using Prism 7 (GraphPad, USA).

## Results

### 3D-CT Position, axial plane

The 3D-CT imaging revealed that 68% of the femoral components had a rotation within ± 3°, whilst the rest showed rotations smaller than − 3°. 43% of the tibial components showed orientations ± 3°, whilst 45% and 12% had axial rotations greater than 3° and smaller than − 3°, respectively.

It was found that in 26% of cases, implants showed relative rotational mismatch within ± 3°. 66% showed internal rotations smaller than − 3° and 8% showed external rotations greater than 3°. Table 3 summarizes all the results, showing median and range values.

**Table 3:** Number of cases, median and range values of orientations of the femoral and tibial components and relative rotational mismatch in the axial plane

	Axial orientation	Number of cases	Median (range) [°]
Femoral component	± 3°	36	− 2 (− 3 to 3)
	> 3°	–	–
	< − 3°	17	− 6 (− 11 to − 4)
Tibial component	± 3°	23	1 (− 3 to 3)
	> 3°	24	8 (4 to 19)
	< − 3°	6	− 9.5 (− 12 to − 6)
Relative rotational mismatch	± 3°	14	− 1 (− 3 to 3)
	> 3°	4	10 (7 to 12)
	< − 3°	35	− 9 (− 22 to − 4)

### 3D-CT Position, coronal plane

87% of the femoral components had orientations within ± 3°, whilst 11% had angulations smaller than − 3° and the 2% showed angulation greater than 3°.

The majority of tibial components showed angulations within ± 3°, whilst 11% and 2% had angulations smaller than − 3° and greater than 3°, respectively.

Considering the tibiofemoral angle, 70% of cases showed orientations within ± 3°, whilst 21% and 9% had angulations smaller than − 3° and greater than 3°, respectively.

Table 4 summarizes all the results, showing median and range values.

**Table 4:** Number of cases, median and range values of orientations of the femoral and tibial components and femorotibial angle in the coronal plane

	Coronal orientation	Number of cases	Median (range) [°]
Femoral component	± 3°	46	0 (− 3 to 3)
	> 3°	1	4 (4)
	<− 3°	6	− 4 (− 7 to − 4)
Tibial component	± 3°	46	− 1 (− 3 to 3)
	> 3°	1	5 (5)
	< − 3°	6	− 4 (− 5 to − 4)
Femorotibial angle	± 3°	37	− 1 (− 3 to 3)
	> 3°	5	5 (4 to 6)
	< − 3°	11	− 4 (− 14 to − 4)

### 3D-CT Position, Sagittal plane

64% of the tibial components showed posterior tibial slopes that were not within 0° to 3° or 5° to 7. 36% had positions of tibial trays within 0° to 3° or 5° to 7°.

Table 5 summarizes all the results, showing median and range values.

**Table 5:** Number of cases, median and range values of orientations of the tibial components in the sagittal plane: negative values mean posterior tibial slope, whilst positive values mean anterior tibial slope

	Sagittal orientation	Number of cases	Median (range) [°]
Posterior Staibilized design	− 3° to 0°	7	− 1 (− 3 to 0)
	Out of − 3° to 0°	6	− 4.5 (− 9 to − 2, 4)
Cruciate Retaining design	− 7° to − 5°	12	− 5 (− 7 to − 5)
	Out of − 7° to − 5°	28	− 3 (− 12 to − 8, − 4 to 2)

### Analysis of polyethylene surfaces

**Fig. 6:**
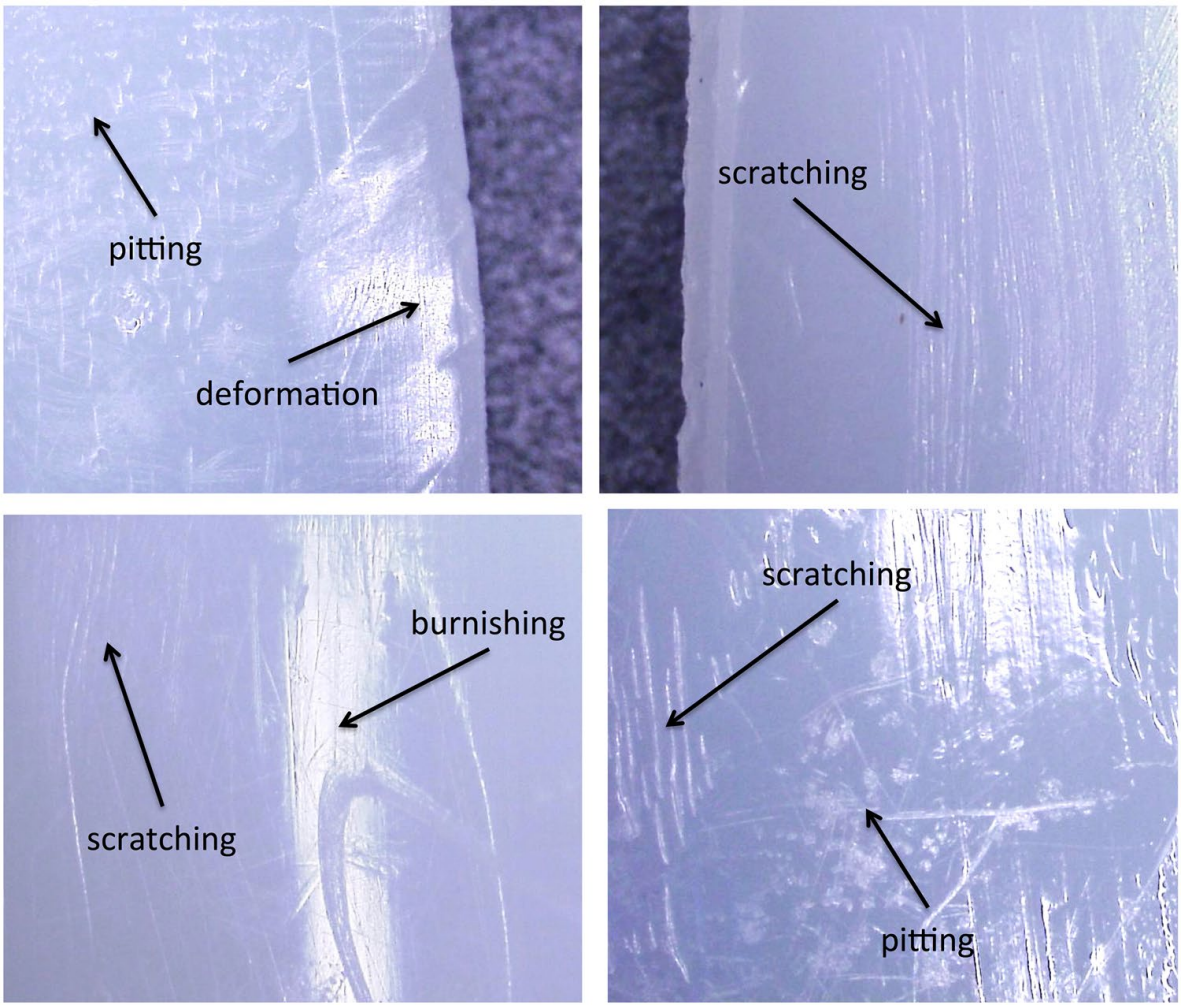
Different modes of surface degradation found on both the articular and backside polyethylene surfaces, including scratching, pitting, burnishing and deformation, at magnification 20X

The most common modes of surface degradation were scratching, pitting and burnishing, whilst deformation and delamination were rare (Fig. 6). The median total Hood score (range) was 39 (17–72). There was no significant difference (*p* = 0.5459) between lateral and medial compartments (defined by the sum of 0–3 and 4–7 sections, respectively, in a right tibial insert; viceversa in a left tibial insert), that showed mean (range) damage scores of 19 (7–39) and 20 (8–41), respectively.

### Correlations

**Fig. 7:**
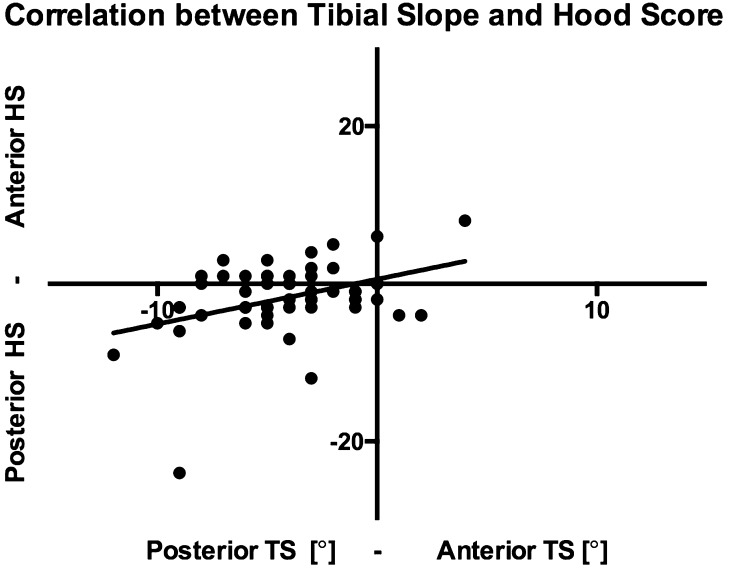
Graph showing the correlation between tibial slope and Hood score. Posterior tibial slope is associated to higher Hood scores in the anterior side of the polyethylene, whilst anterior tibial slope is associated to higher Hood scores in the posterior side of the polyethylene

There was a significant correlation between tibial slope and damage score on polyethylene tibial inserts: posterior tibial slope was associated with higher Hood scores on the anterior areas of the polyethylene, Fig. 7. Table 6 shows further data.

**Table 6:** Correlation between implant orientation and Hood score in all the anatomical planes and differences in Hood scores between normal and suboptimal orientation

		Correlation between orientation and Hood score in expected areas?	Significant difference in the Hood score between normal and suboptimal orientation?
Axial plane	Axial mismatch	n.s. *r* = 0.02978; *p* = 0.8826	n.s. *p* = 0.3042
Coronal plane	Tibiofemoral angle	n.s. *r* = − 0.1168; *p* = 0.4051	n.s. *p* = 0.0964
Sagittal plane	Tibial slope	Significant *r* = − 0.2856; *p* = 0.0382	PS → n.s. *p* = 0.1678 CR → n.s., *p* = 0.0851

*n.s.* not significant

**Fig. 8:**
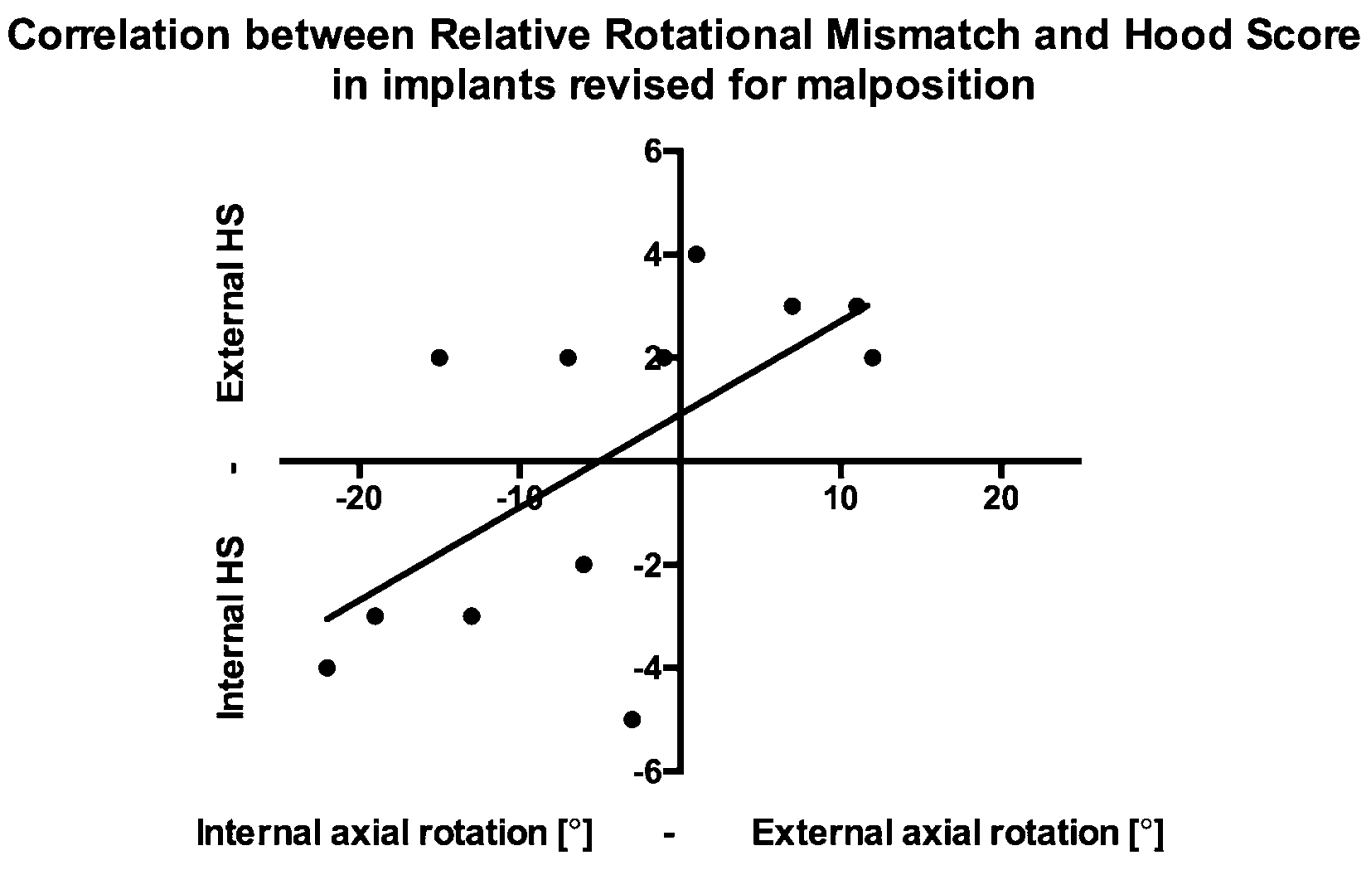
Graph showing the correlation between relative rotational mismatch and Hood score in implants revised for malposition. Internal relative mismatches are associated to higher Hood scores on the posterior compartment in the medial side and on the anterior compartment in the lateral side, while external ones were associated with higher Hood scores on the posterior compartment in the lateral side and on the anterior compartment in the medial side

Separate analyses of the group revised for malposition revealed a significant correlation between implants’ position in the axial plane and damage score on polyethylene tibial inserts, Fig. 8: internal rotation mismatch was associated with higher Hood scores on the posterior compartment in the medial side and on the anterior compartment in the lateral side, while external ones were associated with higher Hood scores on the posterior compartment in the lateral side and on the anterior compartment in the medial side, Table 7.

**Table 7:** Correlation between implant orientation and Hood score in all the anatomical planes in implants revised for malposition

	Correlation between orientation and Hood score in expected areas?
Relative rotational mismatch	Significant *r* = 0.6537, *p* = 0.0240
Femorotibial angle	n.s. *r* = − 0.02154, *p* = 0.8714
Posterior tibial slope	n.s. *r* = − 0.1485, *p* = 0.5814

*n.s.* not significant

There was a significant difference in total Hood score between implants with short (≤ 2 years) and long (> 2 years) implantation time.

**Fig. 9:**
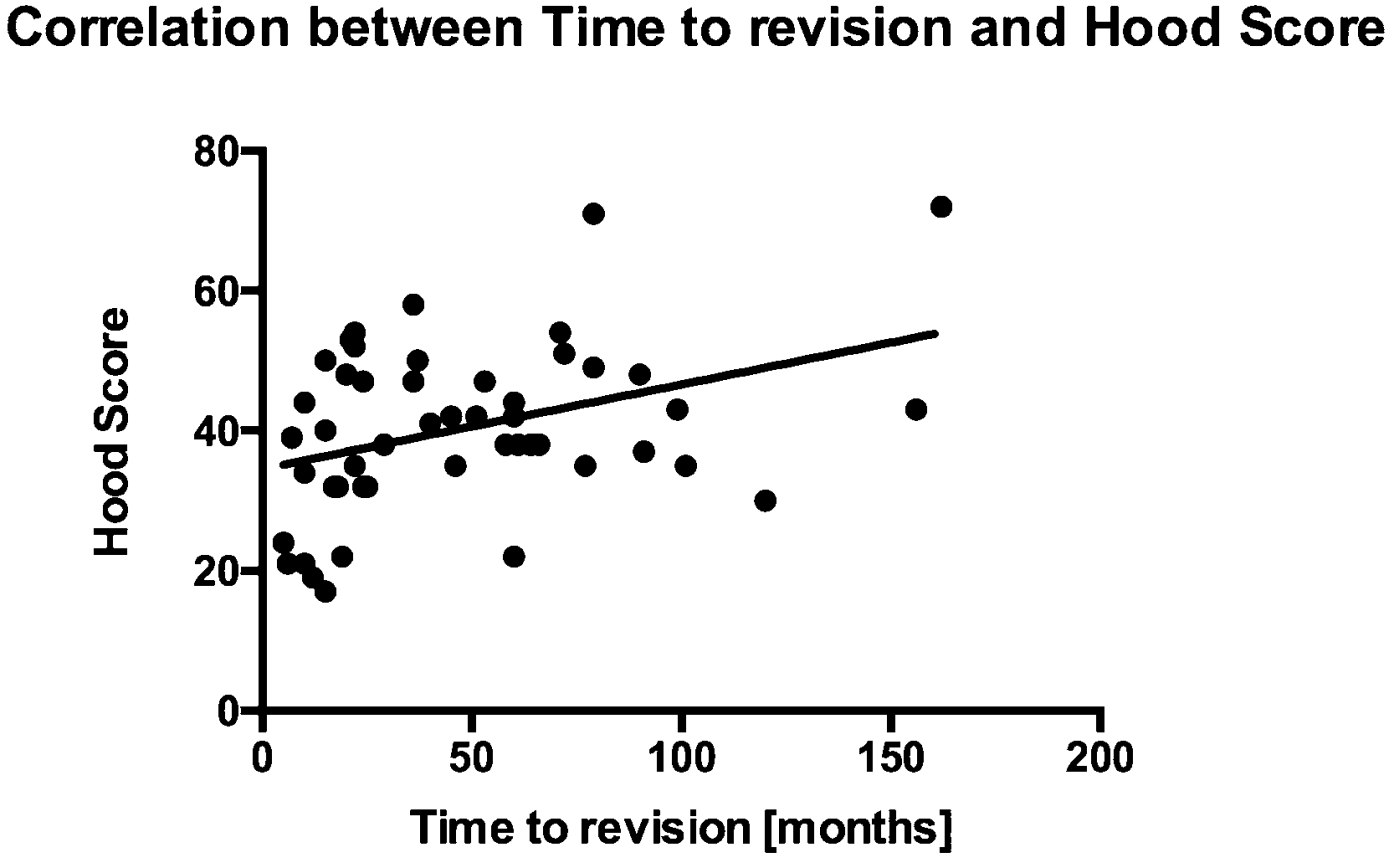
Graph showing the correlation between time to revision and hood score. Retrieval implanted for longer period show higher total Hood scores

There was a significant correlation between time to revision and total Hood score, Fig. 9.

## Discussion

The most important finding of the present study was a significant correlation between relative rotational mismatch and the severity and location of polyethylene damage in implants revised for malposition. There was also a significant correlation between the tibial slope in the sagittal plane, and amount and location of surface damage, but no significant correlation was found between implant orientation and surface damage in polyethylene tibial inserts in both the axial and coronal planes. Moreover, a significant correlation was found between time to revision and hood score. These findings support a multifactorial aetiology of polyethylene wear: implant position alone cannot explain all complex mechanisms involved in the generation of surface damage in polyethylene, especially in early revised implants. This is the first retrieval study to use 3D-CT pre-revision images to measure implant position and correlate findings with results from polyethylene surface damage inspection.

3D-CT-measured implant orientation revealed that the majority of both femoral and tibial components showed optimal orientations in the axial plane, with few cases of excessive internal and external rotations of femoral and tibial components, respectively. Calculations of the relative rotational mismatch revealed that the majority were internally rotated. Regarding the coronal plane, both the femoral and tibial showed mainly optimal orientation, as well as the tibiofemoral angle. In the sagittal plane, almost the totality of the tibial trays showed suboptimal posterior slope.

The current study was the first to investigate the relationship between wear patterns in retrieved knee replacements and 3D-CT-based computed measurements in all the three anatomical planes. In particular, the orientation in the axial plane is difficult to measure with traditional imaging methods [8]; other research groups have identified position in this orientation using roentgenographic score systems [23], X-ray [18, 24] or planar CT images [12, 27, 31, 32]. Axial rotation was measured with accuracy and repeatability using an innovative 3D-CT imaging technique with a standardized, published method and correlates these measurements with results from retrievals. Interestingly, investigating implants revised only for malposition, 9 out of 12 samples showed severe cases of malposition in the axial plane: internal rotational mismatches had a median value (range) of 10 (1–22) degrees, while external rotational mismatches had a median value (range) of 9 (1–12) degrees. In these cases, a significant correlation with the Hood score was found; this could suggest that only severe malposition can lead to asymmetry in the surface damage of polyethylene tibial inserts.

The main characteristic degradation features found on polyethylene inserts were scratching, pitting and burnishing, with no significant difference between medial and lateral sides. This symmetric wear pattern was in contrast to the predominantly asymmetric patterns found in previous studies [18, 20, 22–24]. This discrepancy might be caused by the subjective nature of the widely used Hood score. Indeed, surface damage was found to be only a moderate predictor of wear in polyethylene [33]. Moreover, our components were revised relatively early with median time to revision of 37 months (5–162). The main reasons for revision were instability, malposition and patella maltracking. It is most likely that all patients had poor clinical function and, therefore, low levels of use. This would imply a lower gait cycle number (steps or joint cycles of use per year) and well below the average of 0.9–1.4 million gait cycles per annum. This relative lack of use may also explain why wear patterns did not correlate with components’ positions in all the anatomical planes. Furthermore, the multifactorial nature of the wear pattern mechanisms has to be taken into consideration: this includes a combination of surgical, implant and patient variables. For example, the precision in determining the rotational alignment during a total knee replacement procedure is crucial; correct component positioning can prevent post-cam impingement and reduce the polyethylene wear [32]. Knee replacement design is an important factor that can influence wear [10, 11, 18, 20–23 27, 18]. It has been shown that a new posterior stabilized design can lead to a reduction in polyethylene surface damage and peg deformation [34]. It has also previously been reported that more active patients show greater wear in polyethylene inserts [35].

A considerable number of limitations have to be considered. First, the evaluation of wear pattern was performed using visual scoring for plastic components. Although previous papers [6, 8, 10, 23, 24] used these methods and demonstrated that they can give an acceptable estimation about the quality of the damage, they may be not accurate enough to measure the amount of wear [33]. Recently, the utility of alternative techniques, such as a coordinate measure machine (CMM), laser and micro-CT scanning [24, 25], was demonstrated. Further analyses involving these types of tool may lead to new findings and results, which in combination with 3D CT might allow us to fully understand the role of components’ orientation in total knee replacements and to define a complete “safe zone” for TKA. Future studies should recruit greater number of patients and include a greater number of implants of a single design.

These results showed the importance of optimizing component position to minimize polyethylene damage.

## Conclusion

This is the first study to use accurate measurements from pre-revision 3D-CT to compare tibial and femoral orientation in all three planes with retrieval findings in 53 total knee replacements. Our results revealed a significant correlation between implant axial alignment, and the severity and location of polyethylene damage in implants revised for malposition as well as between tibial slope and damage score on polyethylene tibial inserts.
